# Effects of Dietary *Rhodotorula mucilaginosa* on Muscle Composition, Serum Biochemical Indicators, Antioxidant Capacity, Lipid Metabolism, and mTOR Signaling Pathway in Red Claw Crayfish (*Cherax quadricanatus*)

**DOI:** 10.3390/biology14081089

**Published:** 2025-08-20

**Authors:** Liuqing Meng, Luoqing Li, Ziyi Ma, Wenyan He, Qin Zhang, Tong Tong, Dapeng Wang, Rui Wang, Huizan Yang, Yongqiang Liu, Yin Huang

**Affiliations:** 1 Guangxi Key Laboratory for Polysaccharide Materials and Modifications, School of Marine Sciences and Biotechnology, Guangxi Marine Microbial Resources Industrialization Engineering Technology Research Center, Guangxi Minzu University, 158 University Road, Nanning 530008, China; mengliuqing@stu.gxmzu.edu.cn (L.M.); liluoqing@stu.gxmzu.edu.cn (L.L.); maziyi@stu.gxmzu.edu.cn (Z.M.); hewenyan@stu.gxmzu.edu.cn (W.H.); zhangqin@gxmzu.edu.cn (Q.Z.); tongtong@gxmzu.edu.cn (T.T.); 2 Guangxi Key Laboratory for Aquatic Genetic Breeding and Healthy Aquaculture, Guangxi Academy of Fishery Sciences, 8 Qingshan Road, Nanning 530021, China; oucwdp@163.com (D.W.); raywongxx@163.com (R.W.); yhzyang@163.com (H.Y.)

**Keywords:** red claw crayfish, red yeast, lipid metabolism, signal pathway activation, gene expression

## Abstract

This study investigated the effects of dietary supplementation with varying levels (CK: 0.0 g/kg; RL: 0.1 g/kg; RM: 1.0 g/kg; RH: 10.0 g/kg) of *Rhodotorula mucilaginosa* on muscle composition, serum biochemical indicators, antioxidant capacity, lipid metabolism, and the mTOR signaling pathway in red claw crayfish (*Cherax quadricarinatus*). Results showed that, compared to CK, treatment groups had higher muscle crude protein, fat, leucine, histidine, arginine, and essential amino acids (*p* < 0.05), and lower saturated fatty acids (*p* < 0.05). Treatment groups also exhibited increased activities of alkaline phosphatase, acid phosphatase, superoxide dismutase, catalase, glutathione S-transferase, lysozyme, albumin, total protein, and antioxidant capacity (*p* < 0.05), with reduced activities of aspartate aminotransferase, alanine aminotransferase, lactate dehydrogenase, and triglycerides (*p* < 0.05). In the hepatopancreas, treatment groups showed significant downregulation of AMP-activated protein kinase α, β, and γ, and carnitine palmitoyltransferase 1 genes (*p* < 0.05). Conversely, genes involved in lipid anabolism (peroxisome proliferator-activated receptor γ, acetyl-CoA carboxylase, fatty acid synthase, sterol regulatory element-binding protein, protein kinase B, and mammalian target of rapamycin 1 and 2) were upregulated (*p* < 0.05). In conclusion, *R. mucilaginosa* supplementation affects muscle composition, lipid metabolism, and mTOR signaling. The optimal dose is 1.0 g/kg.

## Simple Summary

Red claw crayfish (*Cherax quadricarinatus*) is an important aquatic species whose health and quality depend largely on diet. To assess the benefits of *Rhodotorula mucilaginosa*, we studied its effects on muscle composition, blood parameters, antioxidant capacity, and lipid metabolism. We tested four feed formulations containing 0, 0.1, 1.0, and 10.0 g/kg of the yeast. Results showed that *R. mucilaginosa* improved the crayfish’s nutritional profile. Muscle protein, beneficial fatty acids, and essential nutrients such as amino acids and minerals increased. Blood analysis revealed higher levels of immune-boosting and antioxidant substances. The yeast also positively affected lipid metabolism and mTOR pathways. The optimal dose was found to be 1.0 g/kg. These findings support the development of more sustainable and nutritionally enhanced feeds for crayfish farming.

## 1. Introduction

Aquatic animals have a high demand for lipids, and the balance of lipid metabolism is directly related to their growth performance, immunity, and environmental adaptability [1]. As a primary source of energy, lipids play a crucial role in essential biological functions, including the synthesis of hormones, the regulation of the immune system, and signal transmission within cells [2]. They function not only as essential precursors for the synthesis of steroid hormones, which influence growth and reproductive processes, but also play a key role in preserving immune balance by modulating the activity and functionality of immune cells, including macrophages and T lymphocytes [3]. However, environmental stress can lead to disruptions in lipid metabolism, which in turn can cause serious problems such as liver dysfunction, impaired immune responses, and delayed growth and development, as seen in studies on juvenile turbot (*Scophthalmus maximus* L.) exposed to high temperatures [4]. In this complex physiological process, the mTOR signaling pathway, as a core regulatory pathway for cell growth, metabolism, and energy balance, regulates protein synthesis, lipid metabolism, and cell growth by integrating nutritional, energy, and growth factor signals [5]. When nutrient levels are adequate and lipid availability is optimal, the mTOR signaling pathway becomes activated. This activation leads to increased expression of fatty acid synthase genes and improves the activity of fatty acid transport proteins, helping to preserve intracellular lipid homeostasis in support of cell growth and essential physiological processes [6].

The red claw crayfish (*Cherax quadricarinatus*), a species of significant economic value in China, relies heavily on the nutritional quality of its feed for healthy development [7]. Since red claw crayfish cannot synthesize essential fatty acids like EPA and DHA de novo, it must rely on its feed to obtain these fatty acids and meet its physiological needs [8]. This dependency highlights the crucial role of lipid metabolism balance in the growth, molting, and immune regulation of crustaceans [9]. Once lipid metabolism becomes abnormal, crustaceans often face problems such as slow growth and molting failure [10].

At present, research on using probiotics to regulate animal lipid metabolism and improve body health has been attracting increasing attention. Dawood et al. found that adding probiotics *Lactobacillus* spp. to feed can effectively regulate the lipid metabolism of tilapia (*Oreochromis niloticus*) by regulating gut microbiota and host metabolism, which not only reduces liver fat deposition but also optimizes the fatty acid composition, thereby promoting the healthy growth of fish [11]. The study by Wang et al. also showed that adding the probiotic *Lactobacillus casei* to feed can improve the effectiveness of high-density lipoprotein cholesterol, reduce the levels of total cholesterol, triglycerides, and low-density lipoprotein cholesterol in the serum and liver tissues of largemouth bass (*Micropterus salmoides*), promote lipid metabolism, lower the blood lipid of largemouth bass, and help the healthy growth of fish [12].

Probiotics play crucial roles in antioxidant defense and immune regulation systems. During metabolic processes in aquatic organisms, homeostasis is maintained through a dynamic equilibrium between reactive oxygen species (ROS) generation and elimination, wherein the antioxidant enzyme defense system plays a pivotal role in reducing and neutralizing ROS [13]. Disruption of this ROS balance accelerates intracellular lipid peroxide formation, subsequently inducing oxidative damage [14]. Probiotics can mitigate oxidative stress damage to organisms by enhancing antioxidant enzyme activity and scavenging free radicals, thereby preserving physiological health [15]. Total antioxidant capacity (T-AOC) serves as an indicator of an organism’s comprehensive antioxidant capability, encompassing the collective action of all antioxidants (e.g., vitamins, enzymes, polyphenolic compounds), and is utilized to assess overall antioxidant status [16]. Superoxide dismutase (SOD) and catalase (CAT) are essential intracellular antioxidant enzymes that scavenge oxygen free radicals, converting superoxide radicals into non-toxic oxygen molecules and water, to maintain normal physiological functions [17]. Concurrently, probiotic-mediated immunomodulation is critically important, with numerous studies documenting yeast applications in aquaculture, such as evidence demonstrating that yeasts enhance antioxidant capacity and immunity in Pacific white shrimp (*Litopenaeus vannamei*), thereby promoting growth [18].

As an emerging probiotic additive, *Rhodotorula mucilaginosa* has been confirmed to possess multifaceted physiological functions and demonstrates unique advantages in regulating lipid metabolism compared to *Lactobacillus* spp., which primarily function by modulating gut microbiota balance and influencing host metabolic pathways; not only can it provide the organism with abundant nutrients, but it can also remodel energy metabolism and immune homeostasis by regulating the expression of lipid metabolism-related genes [19]. *Rhodotorula mucilaginosa* synthesizes bioactive carotenoids that enhance antioxidant capacity and immune function, critically improving health status and stress resistance in aquatic organisms [20]. Simultaneously, yeast-based probiotics have demonstrated promising application potential in aquaculture; for instance, yeast solid-state fermentation products and yeast cultures can significantly downregulate genes associated with lipid synthesis in the liver of zebrafish (*Danio rerio*), alleviate high-fat-diet-induced hepatic inflammation, and maintain organismal health through the regulation of lipid metabolism [21]. As a member of the yeasts, *Rhodotorula mucilaginosa* is also anticipated to play a positive role in aquaculture. Moreover, additional research demonstrates that Torularhodin—a β-carotenoid analog and bioactive component of *Sporidiobolus paradoxus*—functions as a PPAR-γ ligand; engaging in lipid metabolism via the PPAR signaling pathway; modulating key gene expression to enhance adipocyte differentiation and fatty acid uptake; while concurrently suppressing lipolysis [22]. Although the impact of *Rhodotorula mucilaginosa* on lipid metabolism in red claw crayfish has not been systematically investigated, and its capacity to produce bioactive compounds analogous to other yeasts remains unverified, the established physiological functionalities of this yeast, combined with foundational research on yeast-derived probiotics, substantiate its selection as a feed additive with significant research merit and translational potential.

Based on the above studies, this study selected *R. mucilaginosa* as a feed additive to investigate its effects on muscle composition, hemolymph biochemical indices, lipid metabolism, and the mTOR signaling pathway in red claw crayfish. The experimental results will provide a scientific basis for the development of efficient and environmentally friendly feed additives.

## 2. Materials and Methods

### 2.1. Experimental Diets

This research utilized freeze-dried *R. mucilaginosa* powder (≥1 × 10^10^ CFU/g, stored at −20 °C), provided by Guangzhou Xinhaili Biotechnology Co., Ltd. (Zhanjiang, China). The nutritional profile of the powder, on a wet weight basis, included 81.17% water content, 9.27% crude protein, 4.45% crude fat, 4.20% total triglycerides, and 1.30% β-glucan, along with 1.40 mg/kg of β-carotene, 1.00 mg/kg of astaxanthin, and 172.00 mg/kg of vitamin E.

Previous to the experimentation trial, a safety assessment was elaborated following Kang et al.’s protocol [23], utilizing healthy juvenile red claw crayfish (initial body weight 1.10 ± 0.09 g, length 3.08 ± 0.12 cm) purchased from the Nanning Breeding Base of Guangxi Academy of Fishery Sciences (Nanning, China). The red claw crayfish were randomly allocated into six groups (10 individuals/group) and reared in recirculating aquaculture systems under controlled conditions: temperature 26 ± 0.5 °C, ammonia nitrogen < 0.25 ppm, dissolved oxygen 4.7–6.2 mg/L, nitrite 0.01 ppm, nitrate < 10 ppm, pH 7.6 ± 0.2, with a 10 h photoperiod. The tanks measured 60 × 40 × 40 cm with a water flow rate of 2 L/min and a salinity of 0.5%. The treatment groups maintained *R. mucilaginosa* concentrations of 1 × 10^8^ CFU/mL in water, while control groups received no supplementation, with three replicates per group. During the 7-day trial, growth and feeding behavior were monitored daily, with one-third water exchange, bacterial supplementation (treatment groups only), scheduled feeding, and thorough removal of residual feed and feces before/after feeding.

Based on Yang et al.’s findings [24] and safety evaluation data (1 × 10^8^ CFU/mL), four isoproteic (35% CP) and isolipidic experimental diets were formulated with different inclusion levels (Table 1). The preparation protocol involved initially grinding basal feed (Guangdong Hengxing Feed Co., Ltd., Zhanjiang, China) through a 60-mesh sieve (250 μm pore size) to ensure particle uniformity, followed by precise division into four aliquots supplemented with 0 g/kg (Control group, CK), 0.1 g/kg (Low-concentration *R. mucilaginosa* group, RL), 1.0 g/kg (Middle-concentration *R. mucilaginosa* group, RM), and 10.0 g/kg (High-concentration *R. mucilaginosa* group, RH) *R. mucilaginosa* powder, respectively. The components were thoroughly blended for 15 min in a Hobart N50 mixer (Hobart Corporation, Troy, OH, USA) to achieve a consistent mixture. Sterile distilled water, accounting for 40% of the total weight, was then incorporated to create a dough-like consistency, which was subsequently processed into pellets using a Labtech LT-100 pellet mill (Labtech Engineering Co., Ltd., Samut Prakan, Thailand). The pellets, with a uniform diameter ranging from 1.00 to 1.50 mm, were air-dried in an oven (Memmert UN55, Memmert GmbH, Büchenbach, BY, Germany) at a temperature of 30 °C until their moisture level dropped below 10%. The resulting feed was preserved in sealed bags at −20 °C, and new batches were prepared on a weekly basis. To ensure bacterial stability during storage, the viability of live *R. mucilaginosa* in the feed was assessed weekly by plate counting. Detailed nutritional compositions are presented in Table 1.

To ensure the stability of live *R. mucilaginosa* during storage, the viability of the *R. mucilaginosa* in the feed was monitored weekly using standard plate counting throughout the 56-day experimental period [25]. Specifically, 1.0 g feed samples were vortexed with 9.0 mL sterile saline to prepare primary suspensions. Ten-fold serial dilutions were performed to achieve 10^3^–10^8^ concentration gradients. From each dilution, 0.1 mL aliquots were spread on nutrient agar plates in triplicate. All plates were incubated anaerobically at 35 °C for 48 h. Colony counts were recorded post-incubation using a colony counter. Viable counts were calculated as


$$\mathrm{The}\;\mathrm{number}\;\mathrm{of}\;\mathrm{viale}R.mucilaginosa=B\times \frac{C}{10}\times f$$

In the above formula, B is the total number of plate colonies on nutrient agar medium, C is the number of *R. mucilaginosa* colonies identified from 10 colonies, and f is the dilution multiple.

Results showed that viable counts remained stable in each treatment group: 0.89 ± 0.09 × 10^6^ CFU/g (RL group), 0.97 ± 0.12 × 10^7^ CFU/g (RM group), and 0.92 ± 0.16 × 10^8^ CFU/g (RH group), with no significant fluctuations observed during storage. No viable *R. mucilaginosa* was detected in the control group (CK group). This confirmed that the viability of *R. mucilaginosa* was maintained under −20 °C storage conditions, ensuring the accuracy of the experimental doses across the treatment groups.

### 2.2. Experimental Animals and Husbandry Management

Healthy juvenile red claw crayfish, weighing 0.13 ± 0.06 g on average with an average body length of 0.58 ± 0.02 cm, were obtained from the Nanning Breeding Base affiliated with the Guangxi Academy of Fishery Sciences (Nanning, China) for use in this study. Before the start of the experiment, the crayfish were acclimated for a period of 7 days in a recirculating water system within the laboratory environment to ensure they adapted to the experimental conditions and achieved physiological stability. During acclimatization, the crayfish were fed a basal diet daily, and their health status was closely monitored. After acclimatization, healthy juveniles with uniform size, no physical damage, active behavior, and in the intermolt stage were selected for subsequent experiments. All experimental procedures were conducted by the Laboratory Animal Ethics Guidelines of Guangxi Minzu University, Nanning, China (Approval No. GXUN 2023-018).

The formal experiment was carried out in a static rearing system with tanks measuring 1 m × 2 m × 1 m (L × W × H), filled with fresh water to a depth of 70 cm (useful area of 1.4 m^2^). Each tank contained sixteen PVC pipes (75 cm in diameter and 40 cm in length) to serve as shelters, offering hiding spots and minimizing stress. Continuous aeration was provided through oxygen stones, while aquatic plants (*Eichhornia crassipes*) were added to each rearing tank, with four plants per tank, to replicate a natural ecosystem, promoting higher survival rates and improving dissolved oxygen levels. During the experimental period, water conditions were carefully controlled: temperature at 26 ± 0.5 °C, ammonia nitrogen below 0.25 ppm, dissolved oxygen ranging from 4.7 to 6.2 mg/L, nitrite at 0.01 ppm, nitrate under 10 ppm, and pH maintained at 7.6 ± 0.2. Additionally, a 10 h light cycle was implemented to reflect the natural habitat of red claw crayfish.

The experiment consisted of four groups: the CK group (0 g/kg) and three *R. mucilaginosa*-supplemented groups (RL, RM, and RH), with three replicates per group (totaling 12 tanks). Each tank was stocked with 50 juveniles (600 crayfish in total), resulting in a stocking density of 35.71 crayfish/m^3^. The rearing period lasted 56 days. Daily observations were conducted to record growth performance and mortality, allowing timely adjustments in husbandry practices. Following established protocols [26], the daily feeding ratio was set at 5% of body weight, divided into two feedings (08:30 and 18:30), with each feeding administered in two rounds (30 min intervals), and adjusted based on actual consumption to ensure sufficient intake, with 50% of the total feeding provided at each feeding time. Every morning after feeding, residual feed and feces were promptly removed, and one-third of the water volume was exchanged to maintain water quality.

### 2.3. Sample Collection

After the experimental period, all red claw crayfish were fasted for 24 h before sample collection. First, an anticoagulant solution was prepared with glucose (20.5 g/L), sodium citrate (8 g/L), and sodium chloride (4.2 g/L), adjusted to pH 7.5, and then pre-chilled for later use. From each experimental replicate, 12 red claw crayfish were randomly selected, rinsed with sterile physiological saline, and then anesthetized in an ice-water mixture (0 °C) for 10 min to achieve immobilization by freezing. Using a 2.5 mL disposable syringe, 300 μL of pre-chilled anticoagulant was aspirated, and hemolymph was slowly drawn from the ventral sinus at the base of the first abdominal segment. The hemolymph was gently transferred into a 1.5 mL centrifuge tube and centrifuged at 1000× *g* for 10 min at 4 °C. The supernatant was aliquoted into fresh centrifuge tubes and immediately stored at −80 °C for subsequent analysis of hemolymph biochemical indicators and antioxidant enzyme activities (N = 12). After hemolymph collection, the crayfish were transferred to a laminar flow hood for sterile dissection. Abdominal muscle tissue was excised from ten crayfish per group. Muscle samples from five randomly selected crayfish were pooled into a sterile centrifuge tube, flash-frozen in liquid nitrogen, and then stored at −80 °C for subsequent muscle composition analysis (N = 5). Muscle samples from the remaining five crayfish were pooled into another sterile centrifuge tube, flash-frozen in liquid nitrogen, and then stored at −80 °C for subsequent analysis of amino acid and fatty acid composition (N = 5). Simultaneously, hepatopancreas tissue was extracted, weighed using an electronic balance (LICHEN, LC-3000A, Hunan, China), and homogenized in sterile physiological saline at a 1:10 (*w*/*v*) ratio. The homogenate was centrifuged at 1000× *g* for 10 min at 4 °C, and the supernatant was aliquoted into sterile centrifuge tubes before being stored at −80 °C (N = 10).

### 2.4. Determination of Muscle Composition and Nutritional Components

Abdominal muscle tissue from five crayfish per tank was pooled as test samples for the determination of conventional muscle composition. Following the method of Liu et al. [27], the nutritional composition of red claw crayfish muscle samples was analyzed in strict accordance with the standard methods of the Association of Official Analytical Chemists (AOAC, 2005). Crude lipid content was determined using the Soxhlet extraction method, with anhydrous ether as the solvent, under continuous extraction at 65 °C for 6–8 h. Crude protein content was measured using the Kjeldahl method, where muscle samples were digested with concentrated sulfuric acid and then subjected to distillation titration in a Kjeldahl nitrogen analyzer (nitrogen-to-protein conversion factor: 6.25). Moisture content was determined by the constant-weight drying method, where samples were dried in a 105 °C oven until a constant weight was achieved (approximately 6–8 h). Ash content was measured using the high-temperature incineration method, where dried samples were incinerated in a muffle furnace (CHY, CHY-M1700, Zhengzhou, Henan, China) at 550 °C for 4–6 h until complete ashing.

Additionally, muscle tissue samples from five crayfish per tank were collected for nutritional composition analysis. Fatty acid composition was determined by extracting lipids with chloroform–methanol (2:1, *v*/*v*), followed by methylation with boron trifluoride-methanol (BF_3_-MeOH), and analyzing fatty acid methyl esters (FAMEs) via gas chromatography with flame ionization detection (GC-FID). Total and free fatty acid contents in muscle were quantified according to China National Standard GB 5009.168-2016 (Determination of fatty acids in foods) [28]. Hydrolyzed amino acid profiles (17 analytes) were analyzed using a Hitachi L-8900 amino acid analyzer after acid hydrolysis (6 mol/L HCl, 110 °C, 24 h), following China National Standard GB 5009.124-2016 (Determination of amino acids in foods) [29].

### 2.5. Determination of Physiological-Biochemical Parameters and Antioxidant Enzyme Activities in Hemolymph

The hemolymph supernatant previously centrifuged and stored at −80 °C was thawed for analysis. According to the method described by Zhang et al. [30], biochemical parameters and antioxidant enzyme activities in the hemolymph of red claw crayfish were measured using the Rayto RT-6100 ELISA analyzer (Shenzhen, China) with reagent kits supplied by Nanjing Jiancheng Bioengineering Institute. Lysozyme (LZM) in hemolymph was measured by turbidimetry, with units of μg/mL. Alkaline phosphatase (AKP) and acid phosphatase (ACP) were measured by microplate enzymatic methods. The unit of AKP was defined as the amount of enzyme that produces 1 mg of phenol per 100 mL hemolymph after 15 min of reaction with substrate at 37 °C (King unit/100 mL). The unit of ACP was defined as the amount of enzyme that produces 1 mg of phenol per 100 mL hemolymph after 30 min of reaction with substrate at 37 °C (King unit/100 mL). Aspartate aminotransferase (AST) and alanine aminotransferase (ALT) were measured by the microplate method, with units of U/L. Lactate dehydrogenase (LDH) was measured by the microplate method, with one unit defined as the production of 1 μmol pyruvate per liter of serum after 15 min of reaction with substrate at 37 °C (U/L). Total protein (TP) was measured by the BCA microplate method (g/L). Albumin (ALB) was measured by the microplate enzymatic method (g/L). Glucose (GLU) was measured by the glucose oxidase method (mmol/L). Triglycerides (TG) were measured by the GPO-PAP enzymatic method (mmol/L). Total cholesterol (T-CHO) was measured by the COD-PAP method (mmol/L). Catalase (CAT) activity was determined by the ammonium molybdate method and expressed in U/mL, where one unit was defined as the amount of enzyme decomposing 1 μmol of H_2_O_2_ per second in 1 mL of hemolymph supernatant. Superoxide dismutase (SOD) activity was assayed using the WST-1 method, with one unit (U/mL) representing the enzyme quantity required to achieve 50% inhibition in the reaction system. Glutathione S-transferase (GST) and glutathione peroxidase (GPX) activities were measured colorimetrically, both reported in U/mL. One GST unit corresponds to a 1 mol/L decrease in reduced glutathione (GSH) per minute per mL of hemolymph supernatant at 37 °C after deducting non-enzymatic reactions. Similarly, one GPX unit was defined as the enzyme causing a 1 mol/L reduction of GSH in the system per 5 min per 0.1 mL of hemolymph supernatant at 37 °C, corrected for non-enzymatic activity. Malondialdehyde (MDA) content in hemolymph was quantified by the thiobarbituric acid (TBA) assay and expressed in nmol/mL. Total antioxidant capacity (T-AOC) of hemolymph was evaluated using the ABTS radical cation decolorization assay, with results in mmol/L. Detailed protocols can be accessed at http://www.njjcbio.com/ (accessed on 8 March 2025).

### 2.6. Determination of Relative Expression Levels of Genes

Following the method of Liu et al. [31], real-time quantitative PCR (RT-qPCR) was used to detect the expression levels of related genes under RNase-free conditions. Specific primers for AMP-activated protein kinase α (*ampkα*), *ampkβ*, *ampkγ*, peroxisome proliferator-activated receptor γ (*pparγ*), adiponectin receptor (*adipor*), carnitine palmitoyltransferase 1 (*cpt1*), sterol regulatory element-binding protein (*srebp*), acetyl-CoA carboxylase (acc), fatty acid synthase (*fas*), protein kinase B (*akt*), mammalian target of rapamycin 1 (*mtor1*), and *mtor2* were designed using Primer Premier 6.0 software based on published gene sequences in GenBank. *β-Actin* was used as the reference gene for normalization, and primers were synthesized by Sangon Biotech (Shanghai, China). The primer sequences are listed in Table 2.

After thawing the hepatopancreas homogenate supernatant stored at −80 °C, total RNA was extracted using the TaKaRa MiniBEST Universal RNA Extraction Kit (TaKaRa, Beijing, China). RNA concentration (30–1000 ng/μL) and purity (A260/A280 ratio of 1.9–2.1) were measured using a NanoDrop-2000 spectrophotometer, and integrity was verified by 1% agarose TAE gel electrophoresis. RNA was reverse transcribed into cDNA using PrimeScript™ RT Master Mix (Perfect Real Time) (TaKaRa, Beijing, China) according to the manufacturer’s instructions. The reaction system (20 μL) consisted of 10 μL 2× Taqman PCR mix, 1 μL each of forward and reverse primers, RNA template, and DEPC-treated ddH_2_O. Reaction conditions were 30 °C for 10 min, 42 °C for 15 min, 95 °C for 5 min, and 5 °C for 5 min.

RT-qPCR was performed using TB Green^®^ Premix Ex Taq™ II (Tli RNaseH Plus) (TaKaRa, Beijing, China) on a LongGene Q2000B real-time PCR system. The reaction system contained 2 μL cDNA, 10 μL 2 × TB Green Premix Ex Taq II, 6.4 μL DEPC water, and 0.8 μL each of forward and reverse primers (final concentration 0.4 μM). Reaction conditions were pre-denaturation at 95 °C for 30 s; 40 cycles of PCR amplification (95 °C for 5 s, 60 °C for 20 s); and melting curve analysis (95 °C for 0 s, 65 °C for 15 s, 95 °C for 0 s). Each sample was analyzed in triplicate to minimize experimental error.

### 2.7. Data Calculation and Statistics Analysis

All experimental data were initially recorded and processed using Microsoft Excel 2019. Statistical analysis was performed using SPSS Statistics 25.0 software. One-way ANOVA was first conducted to determine significant differences among treatment groups. For a more precise comparison between groups, multiple comparisons were performed using the least significant difference (LSD) method. The significance level was set at *p* < 0.05. Statistically significant differences (*p* < 0.05) between groups were indicated by different letters, while non-significant differences (*p* > 0.05) were indicated by the same letters. Results were presented as mean ± standard deviation (Mean ± SD) to show data central tendency and dispersion. GraphPad Prism 8 software was used for the graphical presentation of data. For RT-qPCR data, the relative expression levels of target genes were calculated using the 2^−ΔΔCT^ method [32].

## 3. Results

### 3.1. Muscle Composition and Nutritional Components

As shown in Table 3, compared to CK, the crude protein content in the muscle of red claw crayfish in the RM group was significantly increased (*p* < 0.05), while no significant differences were observed in the RL, RH, and CK groups (*p* > 0.05).

Compared to the CK group, the crude lipid content in the muscle of red claw crayfish in the RM and RH groups was significantly increased (*p* < 0.05), while no significant differences were observed in the RL and CK groups (*p* > 0.05).

No significant differences were observed in the moisture and ash contents in the muscle of red claw crayfish in the treatment groups and the CK group (*p* > 0.05).

The effects of varying levels of *R. mucilaginosa* on the amino acid composition in the muscle of red claw crayfish are presented in Table 4. Among essential amino acids: arginine (Arg) content in treatment groups was significantly higher than in CK group (*p* < 0.05), wherein the RM and RH groups exhibited significantly elevated arginine (Arg) content compared to the RL group (*p* < 0.05); the RM group displayed the highest leucine content, which was significantly greater than that of CK group (*p* < 0.05), yet demonstrated no statistically significant differences relative to the other two treatment groups; histidine content and total essential amino acid levels in both RM and RH treatment groups were significantly higher than those in CK group (*p* < 0.05), moreover, no significant differences in histidine content were detected between the RL group and all other groups (*p* > 0.05); whereas the total essential amino acid content in the RL group was significantly lower than in both RM and RH groups (*p* > 0.05); but showed no significant difference when compared with CK group (*p* > 0.05).

Non-essential amino acids and total amino acid content in muscle tissue of treatment groups manifested an increasing tendency relative to the CK group, although no statistically significant differences were observed among the experimental groups (*p* > 0.05).

The effects of *R. mucilaginosa* on the fatty acid composition in crayfish muscle are presented in Table 5. Saturated fatty acid (∑SFAs) content in the muscle tissue of treatment groups was significantly lower than that in the control group (*p* < 0.05). Specifically, the C14:0 content in the RH group was significantly reduced compared to the CK group (*p* < 0.05), yet showed no significant differences relative to either the RL group or the RM group (*p* > 0.05); whereas stearic acid (C18:0) content across all treatment groups was significantly elevated compared to the CK group (*p* < 0.05), with no statistically significant variations observed among the supplementation groups (*p* > 0.05).

Monounsaturated fatty acid (∑MUFAs) levels in the muscle of treatment groups were significantly higher than those in the CK group (*p* < 0.05). Notably, palmitoleic acid (C16:1n-7) and oleic acid (C18:1n-9) content in both RM and RH groups were significantly greater than in both CK and RL groups (*p* < 0.05). Polyunsaturated fatty acid (∑PUFAs), ∑n-6 PUFAs, and ∑n-3 PUFAs contents in the muscle tissue of the RM and RH groups demonstrated significant increases relative to the CK group (*p* < 0.05). Specifically, linoleic acid (C18:2n-6), arachidonic acid (C20:4n-6), and eicosapentaenoic acid (C20:5n-3, EPA) levels in the RM and RH groups were significantly higher than in the RL group (*p* < 0.05); whereas no significant difference in docosahexaenoic acid (C22:6n-3, DHA) content was observed between the RM and RL groups (*p* > 0.05).

Long-chain polyunsaturated fatty acid (∑LC-PUFAs) content, total DHA+EPA content, n-3/n-6 PUFA ratio, and fatty acid index (∑FAI) in RM and RH groups were all significantly elevated compared to the other two groups (*p* < 0.05), while the DHA/EPA ratio was significantly lower than in the other treatment groups (*p* < 0.05).

### 3.2. Physiological-Biochemical Parameters and Antioxidant Enzyme Activities in Hemolymph

As shown in Table 6, red claw crayfish in all treatment groups exhibited significantly higher AKP and ACP activities than the CK group (*p* < 0.05). In contrast, the activities of AST, ALT, and LDH were significantly reduced in all three treatment groups (*p* < 0.05).

LZM activity was significantly elevated in the RM and RH treatment groups (*p* < 0.05), whereas no significant difference was observed between the RL and control groups (*p* > 0.05). TP content in the hemolymph was significantly increased in all treatment groups (*p* < 0.05), while ALB content was significantly enhanced only in the RM and RH treatment groups (*p* < 0.05).

Regarding energy metabolism, GLU content significantly decreased only in the RM group (*p* < 0.05), and TG levels were significantly lower in all treatment groups compared to the CK group (*p* < 0.05). T-CHO content, however, showed no significant difference among any groups (*p* > 0.05).

The effects of *R. mucilaginosa* on antioxidant enzyme activities in crayfish hemolymph are shown in Table 7. *R. mucilaginosa* supplementation groups enhanced SOD, CAT, GPX, and GST activities as well as T-AOC, while reducing MDA content in hemolymph. Specifically, SOD, CAT, and GST activities, along with T-AOC in treatment groups, were significantly elevated compared to the CK group (*p* < 0.05), with no significant variations observed among supplementation groups (*p* > 0.05).

Furthermore, relative to the CK group, only the RL and RM treatment groups demonstrated significantly enhanced GPX activity and markedly reduced MDA content (*p* < 0.05), whereas no significant differences in GPX activity or MDA content were detected in the RH group (*p* > 0.05).

### 3.3. Lipid Metabolism and mTOR Signaling Pathway

**Figure 1:**
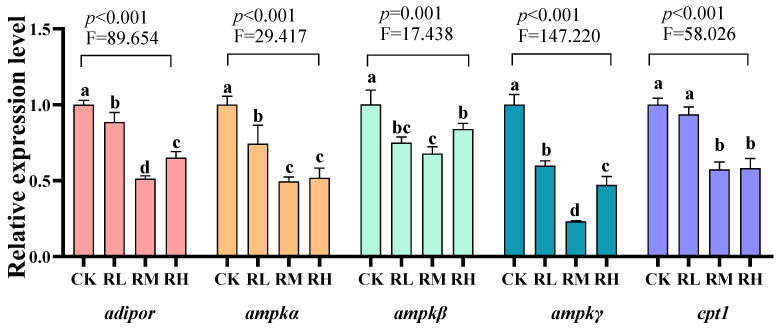
Effects of *Rhodotorula mucilaginosa* on the relative expression levels of adiponectin receptor (*adiopor*), AMP-activated protein kinase α (*ampkα*), AMP-activated protein kinase β (*ampk*β), AMP-activated protein kinase γ (*ampk*γ), and carnitine palmitoyltransferase 1 (*cpt1*) genes in the hepatopancreas of red claw crayfish. All the data are presented as mean ± SD (*n* = 3). Within the same figure, different superscript letters indicate significant differences between the values (*p* < 0.05).

Compared to the CK group, the relative expression levels of *adipor*, *ampkα*, *ampkβ*, and *ampkγ* genes in the hepatopancreas of red claw crayfish in the RL, RM, and RH groups significantly decreased (*p* < 0.05). Compared to the CK group, the relative expression level of *the cpt1* gene in the hepatopancreas of red claw crayfish in the RM and RH groups significantly decreased (*p* < 0.05), while no significant differences were observed in the RL group and CK group (*p* > 0.05), as shown in Figure 1.

**Figure 2:**
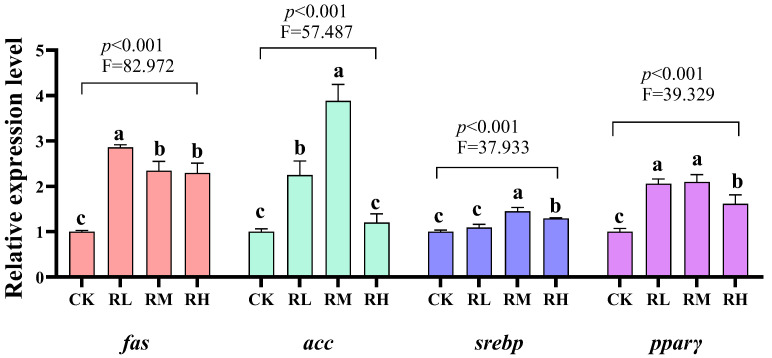
Effects of *Rhodotorula mucilaginosa* on the relative expression levels of fatty acid synthetase (*fas*), acetyl-CoA carboxylase (*acc*), sterol-regulatory element binding proteins (*srebp*), and proliferator-activated receptors γ (*pparγ*) genes in the hepatopancreas of red claw crayfish. All the data are presented as mean ± SD (*n* = 3). Within the same figure, different superscript letters indicate significant differences between the values (*p* < 0.05).

Compared to the CK group, the relative expression levels of *fas* and *pparγ* genes in the hepatopancreas of red claw crayfish in the RL, RM, and RH groups significantly increased (*p* < 0.05). The relative expression level of the *acc* gene in the hepatopancreas of red claw crayfish in the RL and RM groups significantly increased (*p* < 0.05). The relative expression level of the *srebp* gene in the hepatopancreas of red claw crayfish in the RM and RH groups significantly increased (*p* < 0.05), as shown in Figure 2.

**Figure 3:**
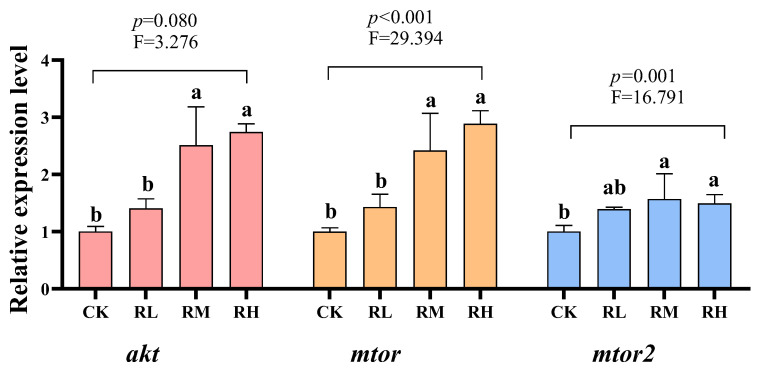
Effects of *Rhodotorula mucilaginosa* on the relative expression levels of protein kinase B (*akt*), mammalian target of rapamycin (*mtor*), and mammalian target of rapamycin 2 (*mtor2*) genes in the hepatopancreas of red claw crayfish. All the data are presented as mean ± SD (*n* = 3). Within the same figure, different superscript letters indicate significant differences between the values (*p* < 0.05).

Compared to the CK group, the relative expression levels of the *akt*, *mtor1*, and *mtor2* genes in the hepatopancreas of red claw crayfish in the RM and RH groups significantly increased (*p* < 0.05), while no significant differences were observed in the RL and CK groups (*p* > 0.05), as shown in Figure 3.

## 4. Discussion

### 4.1. The Effect of Rhodotorula Mucilaginosa on the Muscle Composition and Nutritional Quality in Red Claw Crayfish

Variations in feed composition exert significant influences on the nutritional profile of shrimp meat, which consequently mirrors the metabolic status and growth potential of crustaceans [33]. The nutritional composition of muscle tissue, such as crude protein, crude fat, ash, and moisture content, serves as an important biological indicator for assessing the growth performance and health status of shrimp, as these components are influenced by dietary composition and other environmental factors [34]. The current experimental findings reveal that incorporating varying concentrations of *R. mucilaginosa* into the diet significantly influences the muscle composition of red claw crayfish. Notably, the RM group displayed markedly higher levels of crude protein and lipids in the muscle tissue when compared to the untreated CK group. This suggests that *R. mucilaginosa* can enhance the nutritional value of crayfish. The potential mechanism behind this effect may be attributed to the nutrient-rich composition of *R. mucilaginosa*, which supplies key metabolic precursors, such as amino acids and fatty acids, that activate the mTOR signaling pathway, subsequently promoting increased protein and lipid synthesis [35]. Furthermore, functioning as a probiotic, *R. mucilaginosa* modulates enteroendocrine system functionality to enhance nutrient absorption efficiency, ultimately augmenting protein and lipid retention capacity [27]. Parallel findings documented that probiotic supplementation significantly increased crude protein and ash content in the muscle of Pacific white shrimp (*Litopenaeus vannamei*) [36]. Chen et al. observed that a 1% dietary inclusion of marine red yeast (*Rhodotorula* spp.) elevated crude protein levels in tilapia [37]. Furthermore, this study observed no significant increase in crude muscle protein content in the RH group, a phenomenon potentially attributable to the dose-dependent effects of probiotics. Specifically, the excessively high dose of *R. mucilaginosa* may trigger sustained hyperactivation of immune responses, thereby elevating metabolic burden. This process diverts substantial energy and protein resources—originally allocated for growth—toward immune processes; ultimately reducing energy partitioning for protein synthesis and resulting in the absence of significant crude protein elevation [38].

The types and composition of amino acids are crucial for the growth and development of aquatic organisms, wherein the variety and proportional composition of essential amino acids constitute a core indicator determining the nutritional value of proteins, while the amino acid content in aquatic products serves as a key parameter directly reflecting their nutritional quality [39]. Protein content directly influences muscle growth and nutritional value in crustaceans, and the crux lies in the fact that proteins consist of diverse amino acids, among which the content of essential amino acids (EAAs) represents a vital metric for assessing muscle nutritional value [40]. Results from this study demonstrate that dietary supplementation with *R. mucilaginosa* significantly enhances the content of essential amino acids (arginine, histidine, and leucine) in the muscle of red claw crayfish. Arginine (Arg), as a critical precursor for protein synthesis, not only participates in polyamine biosynthesis, hormonal secretion regulation, and immune function enhancement, but also exerts significant effects on animal growth and cardiovascular health through nitric oxide (NO) generation [41]. Histidine, an essential alkaline amino acid for crustaceans, promotes DNA and protein synthesis by participating in one-carbon metabolism; its imidazole side chain renders it a direct precursor of carnosine and histamine, playing pivotal roles in maintaining homeostasis, regulating muscle pH and osmolality, scavenging reactive oxygen species, and modulating immune functions [42]. Leucine, a key branched-chain amino acid, promotes protein synthesis by activating the mTOR pathway while regulating energy metabolism and glucose homeostasis; it is indispensable for normal growth and reproduction in fish, particularly in stimulating protein synthesis within muscle tissue [43]. Recent research by Xiao et al. [44] revealed that total essential amino acid (TEAA) content in the muscle of greasyback shrimp (*Metapenaeus ensis*) increases with escalating dietary protein levels. Their study indicated that under high-protein feeding regimes, surplus essential amino acids are channeled into catabolism for energy production, whereas low-protein diets induce amino acid deficiencies that subsequently inhibit protein synthesis in shrimp muscle, thereby reducing muscle protein content. In the present experiment, we observed increased essential amino acid content concomitant with elevated crude protein levels in red claw crayfish muscle. Consequently, we posit that this phenomenon may be attributed to the abundant protein constituents in *R. mucilaginosa*, which fulfill the high-protein dietary requirements of red claw crayfish. This suggests that *R. mucilaginosa* not only facilitates the accumulation of essential amino acids but also optimizes the nutritional profile of shrimp muscle by enhancing protein provision. Analogously, Seenivasan et al. [45] reported that *Bacillus subtilis* supplementation elevates arginine, histidine, and leucine content in the muscle of freshwater prawn (*Macrobrachium rosenbergii*).

Fatty acid content and composition in muscle tissue critically determine the nutritional value of aquatic organisms and profoundly influence their flavor profile [40]. Muscle fatty acids are categorized into saturated fatty acids (SFAs) and unsaturated fatty acids (UFAs). In crustaceans, specific fatty acid compositions not only impact their intrinsic health and growth but also determine the quality of their muscle tissues. Research has demonstrated that optimizing feed formulations to reduce saturated fatty acid (SFA) content and increase unsaturated fatty acid (UFA) levels in muscles exerts a proven positive effect on enhancing both the nutritional physiological status and muscle quality of crustaceans themselves [46]. In this experiment, decreased SFA content concomitant with elevated UFA levels in crayfish muscle indicates that *R. mucilaginosa* supplementation enhances muscle quality by modulating fatty acid composition. Studies confirm that MUFAs play vital roles in regulating lipid metabolism, reducing LDL oxidation susceptibility, protecting vascular endothelial function, and attenuating blood hypercoagulability, thereby mitigating cardiovascular disease risks, including coronary heart disease [47]. This study revealed that *R. mucilaginosa* significantly increased palmitoleic acid (C16:1n-7), oleic acid (C18:1n9c), and total MUFA content in crayfish muscle, consistent with alterations in lipid metabolism-related genes, demonstrating its capacity to modulate lipid metabolism via MUFA enrichment. PUFAs markedly enhance aroma and reflect muscle juiciness, with EPA and DHA serving as critical n-3 fatty acids that are key indicators of lipid nutritional value [48]. EPA must be obtained from dietary sources, while DHA is essential for brain development [49]. Higher n-3/n-6 ratios, particularly those rich in EPA and DHA, confer benefits for inflammation control and cardiovascular disease prevention [46]. Additionally, ARA exerts positive effects on growth promotion, immune function enhancement, and antioxidant capacity improvement [50]. This investigation found significantly elevated levels of PUFAs, n-3 PUFAs, linoleic acid (C18:2n6c), ARA (C20:4n6), n-6 PUFAs, EPA (C20:5n3), DHA (C22:6n3), long-chain PUFAs (LC-PUFAs), DHA+EPA, n-3/n-6 ratio, and fatty acid index (FAI) in crayfish muscle, whereas the DHA/EPA ratio was significantly reduced. These findings indicate that *R. mucilaginosa* enhances muscle quality, elevates nutritional value, and boosts immune competence in crayfish. The significantly elevated unsaturated fatty acid (UFA) content observed in crayfish muscle in this study may be attributed to the inherent UFA-rich properties of dietary-supplemented *Rhodotorula mucilaginosa*, which functions as an exogenous UFA source that undergoes digestive absorption and deposition in muscle tissue [51]. Results from Rasool et al. [52] and Mang et al. [53] were consistent with the present experimental outcomes.

### 4.2. The Effect of Rhodotorula Mucilaginosa on the Hemolymph Biochemical Indices and Antioxidant Enzyme Activities in Red Claw Crayfish

As an important body fluid in aquatic animals, the biochemical indices of hemolymph can reflect the health, immune, and metabolic status of the organism [54]. In this experiment, the addition of *R. mucilaginosa* significantly increased the activities of AKP, ACP, and LZM in the hemolymph of red claw crayfish, indicating that it can promote the synthesis of metabolic immune enzymes in crayfish and enhance disease resistance and immunity. This may be related to the presence of β-glucan in *R. mucilaginosa* cells, which binds to high-density lipoproteins and activates the prophenoloxidase system, thereby enhancing the activity of immune-related enzymes [55]. Similar results have been reported in Pacific white shrimp [35].

AST and ALT are key indicators reflecting liver function, and an increase in their activities often suggests hepatopancreas damage or metabolic disorders [53]. In this experiment, *R. mucilaginosa* significantly reduced the activities of ALT and AST, indicating that it can alleviate stress in crayfish and maintain stable metabolic function. This may be attributed to the prebiotic components in yeast activating the Toll-like receptor pathway and enhancing the phagocytic activity of hemocytes to improve hepatopancreas health [56]. A similar effect has been demonstrated in Pacific white shrimp with the use of *Bacillus* T23 [57].

LDH, a key enzyme in glycolysis, exhibits increased activity during organismal stress or tissue damage [58], while blood glucose levels reflect the state of energy metabolism [59]. This experiment showed that *R. mucilaginosa* significantly reduced LDH activity and blood glucose levels in the hemolymph of red claw crayfish. It is speculated that this may be due to astaxanthin in yeast activating the AMPK signaling pathway to regulate glycolysis, consume energy, and produce ATP through glucose metabolism [60].

TP and ALB levels can be used to evaluate the immune status of aquatic animals [61]. In this study, the *R. mucilaginosa* groups exhibited higher TP and ALB levels compared to the CK group, which may be related to the astaxanthin and mannan oligosaccharides in yeast increasing metabolic rates and accelerating protein synthesis [27]. TG is an important energy source for the organism, and fluctuations in T-CHO levels can reflect lipid metabolism in the liver [62]. In this experiment, *R. mucilaginosa* significantly reduced TG levels but had no significant effect on T-CHO, indicating its beneficial role in metabolic activities. This may be attributed to the nutrient-rich composition of *R. mucilaginosa* enhancing lipase activity and improving lipid metabolism [63]. These findings are consistent with the results reported by Liu et al., where the addition of *R. mucilaginosa* to tilapia feed led to increased serum TP and ALB levels and decreased GLU, TG, and T-CHO levels [27].

This study observed significantly enhanced SOD and CAT activities along with elevated T-AOC in crayfish hemolymph, indicating that *R. mucilaginosa* strengthens antioxidant capacity, mitigates oxidative stress, and preserves physiological integrity—effects we attribute primarily to yeast-derived carotenoids; particularly β-carotene and astaxanthin [38]. Carotenoids function as potent antioxidants that effectively scavenge oxygen radicals, attenuate cellular oxidative damage, and modulate SOD/CAT activities [64], consistent with findings reported by Kieliszek et al. [65]. GPX eliminates hydrogen peroxide and other peroxides to protect cells from oxidative injury, while GST facilitates ROS clearance through concerted action with GPX in hydrogen peroxide detoxification [66]. Significantly increased GPX and GST activities observed herein may result from astaxanthin in red yeast maintaining cellular redox homeostasis and reducing ROS levels, thereby indirectly potentiating these enzymatic activities [67]. MDA, as a lipid peroxidation end-product, quantitatively reflects cellular oxidative damage [68]. Our results demonstrated significantly reduced MDA content in the hepatopancreas of *R. mucilaginosa*-supplemented red claw crayfish. This reduction likely stems from β-glucans and mannans in the yeast cell wall forming complexes with Fe^2+^, inhibiting hydroxyl radical (·OH) generation, consequently disrupting lipid peroxidation chain reactions, and decreasing MDA production [69]. Analogous results were reported by Sriphuttha et al. [70], where *Rhodotorula paludigena* enhanced antioxidant and immune capacities in Pacific white shrimp (*Litopenaeus vannamei*). Similarly, Wang et al. documented that selenium yeast supplementation boosts antioxidant capability in Chinese mitten crab (*Eriocheir sinensis*) [71].

### 4.3. Effects of Rhodotorula Mucilaginosa on Lipid Metabolism and mTOR Signaling Pathway in Red Claw Crayfish

Lipid metabolism, serving as a fundamental physiological process governing the synthesis, degradation, and transport of fatty acids, triglycerides, and related compounds, plays a pivotal role in the growth, development, and health maintenance of aquatic species [72]. This study revealed that *R. mucilaginosa* supplementation downregulated the expression of key fatty acid β-oxidation genes (including *ampkα*, *ampkβ*, *ampkγ*, *adipor*, and *cpt1*) in the hepatopancreas of crayfish, suggesting its potential regulatory effects on energy sensing and signal transduction pathways. AMP-activated protein kinase (AMPK), functioning as an energy-sensing kinase, becomes activated under low ATP conditions to restore energy homeostasis by enhancing fatty acid oxidation while suppressing lipid synthesis. The *adipor* potentiates fatty acid uptake and oxidation by transducing adiponectin signals to activate AMPK and its downstream pathways [73]. Carnitine palmitoyl transferase 1 (CPT1), the rate-limiting enzyme for mitochondrial fatty acid β-oxidation, exhibits reduced activity when downregulated, thereby impeding the transport of fatty acyl-CoA into mitochondria and attenuating oxidative processes [74]. The observed gene downregulation may stem from dual mechanisms: primarily, the protein-, polysaccharide-, and carotenoid-rich composition of *R. mucilaginosa* [35] could elevate cellular ATP levels, diminishing AMPK pathway activation and consequently suppressing *ampkα*, *ampkβ*, *ampkγ*, and *cpt1* expression. Alternatively, *R. mucilaginosa* might disrupt adiponectin-receptor binding or AMPK signaling cascades, inhibiting fatty acid oxidation-related signal transmission via *adipor* downregulation [75]. Notably, AMPK in crustaceans operates as a master energy sensor, with its activity and gene expression dynamically responding to environmental fluctuations to modulate energy demands [76]. Thus, *R. mucilaginosa*-induced transcriptional alterations likely reflect an adaptive metabolic reprogramming under energy-replete conditions—curtailing fatty acid oxidation to minimize energy expenditure while augmenting lipid storage to accommodate nutritional shifts.

The study also demonstrated that dietary *R. mucilaginosa* significantly upregulated hepatopancreatic expression of *fas*, *acc*, *srebp*, and *pparγ*, indicating its capacity to stimulate lipogenesis via modulation of core fatty acid synthesis genes. Acetyl-CoA carboxylase (ACC), the rate-limiting enzyme in fatty acid biosynthesis, catalyzes malonyl-CoA formation [77]. Fatty acid synthase (FAS) mediates long-chain fatty acid elongation [78]. Sterol regulatory element-binding proteins (SREBPs), encoded by *srebp*, bind promoter regions of *fas* and *acc* to activate their transcription and coordinate lipid biosynthesis [79]. Peroxisome proliferator-activated receptor gamma (PPARγ), a central regulator of adipogenesis, enhances lipid deposition by transactivating lipogenic enzyme genes [80]. Mechanistically, AMPK pathway activation suppresses *srebp* transcription and ACC enzymatic activity to potentiate fatty acid oxidation while inhibiting synthesis [81]. In this study, *R. mucilaginosa*-mediated AMPK inhibition (via *ampkα/β/γ* downregulation) presumably alleviated AMPKs suppression of *srebp*, enabling its upregulated expression and subsequent initiation of *fas/acc* transcriptional programs. Moreover, bioactive constituents in *R. mucilaginosa* (e.g., carotenoids and polysaccharides) may directly engage PPARγ signaling, activating *pparγ* expression to synergize with SREBP in orchestrating a lipogenic transcriptional network that collectively drives fatty acid synthesis and lipid accumulation [82]. These coordinated gene expression shifts reveal *R. mucilaginosa*’s dual-modulatory mechanism, which simultaneously suppresses fatty acid oxidation pathways while activating lipogenic routes, thereby remodeling crayfish lipid metabolism and ultimately enhancing lipid storage capacity.

Previous studies established the mTOR pathway as a central hub for cellular growth, metabolism, and energy homeostasis, wherein mTOR complex 1 (mTORC1) and mTORC2 integrate nutrient, energy, and growth factor signals to coordinately regulate protein synthesis and lipid metabolism [5]. Specifically, mTORC1 promotes protein biosynthesis via phosphorylation of downstream effectors (e.g., S6K1 and 4E-BP1) while modulating lipid metabolism through transcriptional regulators like sterol regulatory element-binding proteins (SREBPs). mTORC2 participates in cytoskeletal reorganization and metabolic regulation [83]. AKT, a critical upstream mTOR regulator, phosphorylates and inhibits tuberous sclerosis complex 2 (TSC2), thereby relieving its suppression on the small GTPase Rheb to activate mTORC1 [84]. Dietary *R. mucilaginosa*, abundant in proteins and polysaccharides, likely elevates phosphorylated AKT levels via PI3K/AKT pathway activation, subsequently triggering Rheb-mediated mTORC1 stimulation through this mechanism. Activated mTOR signaling enhances ribosomal biogenesis and translation initiation via S6K1 and 4E-BP1 phosphorylation to potentiate protein synthesis. Concurrently, it upregulates SREBP transcriptional activity and downstream *fas*/*acc* expression, synergizing with PPARγ signaling to collectively drive fatty acid biosynthesis and lipid deposition [6]. This mechanism aligns precisely with our observations of upregulated lipogenic genes (*fas*, *acc*, *srebp*, and *pparγ*) and increased muscle crude fat content. *R. mucilaginosa* optimizes nutrient partitioning by leveraging mTOR-mediated dual effects on protein anabolism and lipogenesis, thereby modulating growth and lipid metabolism.

## 5. Conclusions

In conclusion, supplementing the diet with *R. mucilaginosa* has a notable impact on the muscle composition of red claw crayfish, influencing both amino acid and fatty acid profiles, as well as affecting hemolymph biochemical parameters and antioxidant enzyme activities. The supplementation suppresses genes associated with lipid breakdown, enhances those related to lipid synthesis, and modulates key metabolic signaling pathways by inhibiting AMPK while activating mTOR. Based on the findings of this research, the recommended minimum inclusion level of *R. mucilaginosa* is 1.0 g per kg of diet.

## Figures and Tables

**Table 1:** The formulation and composition of the experimental diets for red claw crayfish (g/kg of dried diet).

Ingredients	CK	RL	RM	RH
Fish meal	485.00	485.00	485.00	485.00
Soybean meal	119.30	119.20	118.30	109.30
Sorghum flour	106.50	106.50	106.50	106.50
Wheat flour	139.00	139.00	139.00	139.00
Corn flour	45.00	45.00	45.00	45.00
Soy Lecithin	10.00	10.00	10.00	10.00
Fish oil	15.20	15.20	15.20	15.20
Gelatin	20.00	20.00	20.00	20.00
Calcium carbonate	10.00	10.00	10.00	10.00
Choline chloride	5.00	5.00	5.00	5.00
Mineral premixes ^a^	20.00	20.00	20.00	20.00
Vitamin premixes ^b^	20.00	20.00	20.00	20.00
Vitamin C	5.00	5.00	5.00	5.00
Proximal composition ^c^ (% Dry Matter)				
Dry material	92.56	92.56	92.56	92.56
Ash	7.76	7.76	7.76	7.76
Ethereal extract	7.40	7.40	7.40	7.40
Crude protein	35.20	35.20	35.20	35.20
Crude lipid	7.84	7.84	7.84	7.84
Fiber	3.43	3.43	3.43	3.43

Note: All ingredients used in this study were sourced from Guangdong Hengxing Feed Co., Ltd. (Zhanjiang, China)., and are marketed under the brand name “Hengxing”. ^a^ Mineral premixes (mg/kg): KCl, 0.5; MgSO_4_·7H_2_O, 0.5; ZnSO_4_·7H_2_O, 0.09; MnCl_2_·4H_2_O, 0.0234; CuSO_4_·5H_2_O, 0.005; KI, 0.005; CoCl_2_·2H_2_O, 0.0025; Na_2_HPO_4_, 2.37. ^b^ Vitamin premixes (mg/kg): Vitamin B_12_, 0.02; Vitamin A acetate, 5000 IU; Vitamin D_3_, 4000 IU; α-tocopherol acetate, 100 IU; menadione, 5; thiamine HCl, 60; riboflavin, 25; pyridoxine HCl, 50; folic acid, 10; dl-capantothenic acid, 75; nicotinic acid, 5; biotin, 1; inositol, 5. ^c^ These values are calculated from the formulation and do not represent actual measured values.

**Table 2:** Primer sequences for RT-qPCR in red claw crayfish.

Gene	Primer Sequence (5′→3′)	Amplicon Size (bp)	Tm (°C)	Gene Bank
*fas* ^1^	F: TTGACTTCAAAGGTCCCAGC	126	58	XM_053782425.1
	R: GGTTAGTACCTCCCACCACT			
*acc* ^2^	F: GTCAGGAAGTTTGGAGGCAA	136	58	XM_053793522.1
	R: TGAAATGAAAGGCACGGTCA			
*cpt1* ^3^	F: ACACCTGCCTATTGGTTGGG	142	60	XM_053797361.1
	R: CTCAAGTCTTGGTGGGCTCC			
*adipor* ^4^	F: AACGACAGTGTACGCCTCTG	155	60	XM_053787771.1
	R: TGAGGAAAGGCAGCGTGAAG			
*ampkα* ^5^	F: TTAAGTGGTGACCGAGGAGT	186	60	XM_053773531.1
	R: AAACCTCGCTCATGATGTCC			
*ampkβ* ^6^	F: ATGATGTAGGCTCGCAGAAC	108	58	XM_053796745.1
	R: GCGGCTTGTCCTTATTGTTG			
*ampkγ* ^7^	F: CTGACCCCTTCCTGGAAAAC	97	58	XM_053773266.1
	R: AGGTCATAGGTGTGGTGGAA			
*ppar-γ* ^8^	F: CGGATGGTGGTGGAGGAGTAGG	81	63	XM_053785396.1
	R: CCCAGGAGCCAACGACAACAC			
*srebp* ^9^	F: GTCTTCCTGGTGGGTCTCCTCTAC	85	63	XM_053788865.1
	R: CGACTTCCGCCTCACTCTCAATG			
*akt* ^10^	F: GGAAGCAGCAGCAGCAGTGG	136	63	XM_053793380.1
	R: TGGAAGTTCGGCGGTCAATCATC			
*mtor1* ^11^	F: GGAGGAGGAGGAAGAAGAGGAAGC	142	64	XM_053785700.1
	R: GCACAACACCAGAGCCACACTC			
*mtor2* ^12^	F: GTAGCGGCACTGCGGGTTG	175	64	XM_053771218.1
	R: TTGGTGGGAGGTGGCTGGTG			
*β-actin* ^13^	F: ATCACTGCTCTGGCTCCTGCTACC	148	60	XM_053800817.1
	R: CGGACTCGTCGTACTCCTCCTTGG			

Note: F: Forward primer. R: Reverse primer. ^1^ *fas*: Fatty acid synthetase; ^2^ *acc*: acetyl-CoA carboxylase; ^3^ *cpt1*: Carnitine palmitoyltransferase 1; ^4^ *adiopor*: Adiponectin receptor; ^5^ *ampkα*: AMP-activated protein kinase α; ^6^ *ampkβ*: AMP-activated protein kinase β; ^7^ *ampkγ*: AMP-activated protein kinase γ; ^8^ *pparγ*: Proliferator-activated receptors γ; ^9^ *srebp*: Sterol-regulatory element binding proteins; ^10^ *akt*: Protein kinase B; ^11^ *mtor1*: Mammalian target of rapamycin 1; ^12^ *mtor2*: Mammalian target of rapamycin 2; ^13^ *β-actin*: Non-regulated reference gene.

**Table 3:** The effects of *Rhodotorula mucilaginosa* in crayfish diets on the muscle composition.

Index	CK	RL	RM	RH	F-Value	*p*-Value
Moisture (%)	80.67 ± 1.79	80.23 ± 1.52	78.03 ± 1.41	79.57 ± 1.27	1.757	0.233
Ash (%)	5.38 ± 0.15	5.41 ± 0.13	5.41 ± 0.08	5.40 ± 0.12	0.040	0.989
Crude protein (%)	9.84 ± 0.23 ^b^	10.37 ± 0.52 ^a,b^	11.37 ± 0.55 ^a^	10.90 ± 0.25 ^a,b^	7.623	0.010
Crude lipid (%)	1.28 ± 0.03 ^b^	1.30 ± 0.04 ^b^	1.41 ± 0.05 ^a^	1.39 ± 0.03 ^a^	8.475	0.007

Note: All the data are presented as mean ± SD (*n* = 3). Within the same row, different superscript letters indicate significant differences between the values (*p* < 0.05).

**Table 4:** The effects of *Rhodotorula mucilaginosa* in crayfish diets on the amino acid composition of muscle (g/100 g dry matter).

Index	CK	RL	RM	RH	F-Value	*p*-Value
Arginine	10.05 ± 0.07 ^c^	10.18 ± 0.04 ^b^	10.45 ± 0.12 ^a^	10.32 ± 0.04 ^a^	17.268	0.001
Histidine	2.3 ± 0.04 ^b^	2.33 ± 0.03 ^a,b^	2.38 ± 0.03 ^a^	2.39 ± 0.04 ^a^	5.263	0.027
Isoleucine	3.55 ± 0.05	3.58 ± 0.03	3.67 ± 0.09	3.67 ± 0.07	2.669	0.119
Leucine	6.74 ± 0.07 ^b^	6.78 ± 0.09 ^a,b^	6.89 ± 0.03 ^a^	6.85 ± 0.04 ^a,b^	3.138	0.087
Lysine	7.56 ± 0.03	7.59 ± 0.04	7.62 ± 0.06	7.64 ± 0.04	2.121	0.176
Methionine	2.1 ± 0.05	2.14 ± 0.03	2.15 ± 0.02	2.14 ± 0.04	1.270	0.348
Phenylalanine	3.32 ± 0.04	3.29 ± 0.07	3.27 ± 0.07	3.29 ± 0.04	0.517	0.682
Threonine	3.4 ± 0.03	3.4 ± 0.04	3.37 ± 0.06	3.42 ± 0.03	0.793	0.531
Valine	3.91 ± 0.04	3.95 ± 0.03	3.99 ± 0.04	3.97 ± 0.06	1.749	0.234
Total essential amino acids	42.94 ± 0.23 ^b^	43.23 ± 0.26 ^b^	43.79 ± 0.06 ^a^	43.68 ± 0.12 ^a^	13.129	0.002
Alanine	4.53 ± 0.06	4.5 ± 0.06	4.52 ± 0.03	4.17 ± 0.52	1.281	0.345
Aspartic acid	9.04 ± 0.08	9.07 ± 0.09	9.08 ± 0.09	9.09 ± 0.09	0.135	0.937
Glutamic acid	14.91 ± 0.09	14.98 ± 0.11	15.09 ± 0.09	15.01 ± 0.09	1.974	0.196
Glycine	3.76 ± 0.09	3.8 ± 0.08	3.74 ± 0.09	3.85 ± 0.08	1.060	0.418
Proline	3.63 ± 0.06	3.65 ± 0.05	3.65 ± 0.07	3.65 ± 0.05	0.103	0.956
Serine	3.65 ± 0.08	3.72 ± 0.05	3.67 ± 0.05	3.71 ± 0.05	1.088	0.408
Tyrosine	3.77 ± 0.05	3.77 ± 0.03	3.78 ± 0.05	3.77 ± 0.05	0.026	0.994
Total non-essential amino acids	43.3 ± 0.31	43.48 ± 0.14	43.53 ± 0.19	43.26 ± 0.56	0.456	0.721
Total amino acids	86.24 ± 0.53	86.72 ± 0.33	87.32 ± 0.25	86.94 ± 0.58	3.078	0.090

Note: All the data are presented as mean ± SD (*n* = 3). Within the same row, different superscript letters indicate significant differences between the values (*p* < 0.05).

**Table 5:** The effects of *Rhodotorula mucilaginosa* in crayfish diets on muscle fatty acid composition (% of total fatty acids).

Index	CK	RL	RM	RH	F-Value	*p*-Value
C14:0	0.4 ± 0.03 ^a^	0.38 ± 0.02 ^a,b^	0.36 ± 0.02 ^a,b^	0.35 ± 0.03 ^b^	2.568	0.127
C16:0	1.55 ± 0.03	1.53 ± 0.03	1.53 ± 0.03	1.53 ± 0.03	0.615	0.624
C17:0	0.39 ± 0.01	0.38 ± 0.01	0.38 ± 0.01	0.38 ± 0.01	0.800	0.528
C18:0	8.75 ± 0.04 ^a^	8.7 ± 0.02 ^b^	8.68 ± 0.02 ^b^	8.66 ± 0.03 ^b^	6.927	0.013
∑SFAs ^1^	11.1 ± 0.05 ^a^	10.99 ± 0.03 ^b^	10.95 ± 0.06 ^b^	10.93 ± 0.03 ^b^	9.088	0.006
C16:1n-7	4.89 ± 0.04 ^c^	4.99 ± 0.03 ^b^	5.09 ± 0.04 ^a^	5.12 ± 0.04 ^a^	20.829	<0.001
C18:1n-9	25.55 ± 0.13 ^c^	25.99 ± 0.12 ^b^	26.48 ± 0.33 ^a^	26.75 ± 0.24 ^a^	17.251	0.001
∑MUFAs ^2^	30.44 ± 0.14 ^c^	30.98 ± 0.15 ^b^	31.57 ± 0.34 ^a^	31.87 ± 0.28 ^a^	20.807	<0.001
C20:2n-9	0.88 ± 0.01	0.87 ± 0.01	0.88 ± 0.02	0.88 ± 0.01	0.889	0.487
C18:2n-6	14.4 ± 0.04 ^b^	14.47 ± 0.09 ^b^	14.61 ± 0.04 ^a^	14.6 ± 0.05 ^a^	9.125	0.006
C18:3n-6	0.18 ± 0.01	0.18 ± 0.01	0.18 ± 0.01	0.18 ± 0.01	1.222	0.363
C20:3n-6	3.33 ± 0.02	3.33 ± 0.02	3.33 ± 0.02	3.33 ± 0.02	0.111	0.951
C20:4n-6 (ARA ^3^)	0.81 ± 0.01 ^b^	0.81 ± 0.01 ^b^	0.85 ± 0.03 ^a^	0.86 ± 0.02 ^a^	6.838	0.013
C22:4n-6	1.1 ± 0.02	1.09 ± 0.03	1.09 ± 0.04	1.09 ± 0.03	0.190	0.900
∑n-6 PUFAs ^4^	19.82 ± 0.04 ^b^	19.87 ± 0.09 ^b^	20.06 ± 0.03 ^a^	20.06 ± 0.03 ^a^	17.797	0.001
C18:3n-3	3.82 ± 0.06	3.82 ± 0.04	3.77 ± 0.09	3.71 ± 0.04	1.797	0.226
C20:5n-3 (EPA ^5^)	13.35 ± 0.29 ^b^	13.79 ± 0.81 ^b^	15.07 ± 0.28 ^a^	15.31 ± 0.19 ^a^	12.880	0.002
C22:5n-3	1.24 ± 0.03	1.25 ± 0.02	1.26 ± 0.02	1.26 ± 0.02	0.523	0.679
C22:6n-3 (DHA ^6^)	3.68 ± 0.05 ^c^	3.74 ± 0.03 ^b,c^	3.82 ± 0.05 ^a,b^	3.84 ± 0.04 ^a^	8.350	0.008
∑n-3 PUFAs ^7^	22.1 ± 0.37 ^b^	22.6 ± 0.86 ^b^	23.92 ± 0.14 ^a^	24.12 ± 0.19 ^a^	12.680	0.002
∑PUFAs ^8^	42.8 ± 0.39 ^b^	43.34 ± 0.82 ^b^	44.86 ± 0.15 ^a^	45.06 ± 0.18 ^a^	17.018	0.001
∑LC-PUFAs ^9^	23.52 ± 0.31 ^b^	24 ± 0.82 ^b^	25.43 ± 0.21 ^a^	25.68 ± 0.17 ^a^	16.007	0.001
n-3/n-6	1.11 ± 0.02 ^b^	1.14 ± 0.05 ^b^	1.19 ± 0.01 ^a^	1.2 ± 0.01 ^a^	8.611	0.007
DHA+EPA	17.04 ± 0.33 ^b^	17.53 ± 0.83 ^b^	18.89 ± 0.23 ^a^	19.15 ± 0.16 ^a^	14.37	0.001
DHA/EPA	0.28 ± 0.01 ^a^	0.27 ± 0.01 ^a^	0.25 ± 0.01 ^b^	0.25 ± 0.01 ^b^	6.536	0.015
∑FAI ^10^	84.34 ± 0.51 ^b^	85.31 ± 0.96 ^b^	87.37 ± 0.42 ^a^	87.85 ± 0.48 ^a^	21.196	<0.001

Note: All the data are presented as mean ± SD (*n* = 3). Within the same row, different superscript letters indicate significant differences between the values (*p* < 0.05). ^1^ ∑SFAs: Saturated fatty acids; ^2^ ∑MUFAs: Monounsaturated fatty acids; ^3^ ARA: Arachidonic acid; ^4^ ∑n-6 PUFAs: n-6 Polyunsaturated fatty acids; ^5^ EPA: Eicosapentaenoic acid; ^6^ DHA: Docosahexaenoic acid; ^7^ ∑n-3 PUFAs: n-3 Polyunsaturated fatty acids; ^8^ ∑PUFAs: Polyunsaturated fatty acids; ^9^ ∑LC-PUFAs: Long-chain polyunsaturated fatty acids; ^10^ ∑FAI: Fatty acid index.

**Table 6:** The effects of *Rhodotorula mucilaginosa* in crayfish diets on the hemolymph biochemical indices.

Index	CK	RL	RM	RH	F-Value	*p*-Value
AKP 1 (King unit/100 mL)	18.76 ± 0.24 ^c^	20.46 ± 0.38 ^b^	22.17 ± 0.11 ^a^	20.73 ± 0.68 ^b^	11.481	0.003
ACP 2 (King unit/100 mL)	16.10 ± 0.41 ^b^	18.11 ± 0.51 ^a^	18.81 ± 0.29 ^a^	17.71 ± 0.38 ^a^	24.115	<0.001
AST 3 (U/L)	2.51 ± 0.14 ^a^	1.65 ± 0.09 ^b^	1.16 ± 0.21 ^b^	1.40 ± 0.13 ^b^	15.869	0.001
ALT 4 (U/L)	1.65 ± 0.08 ^a^	1.13 ± 0.07 ^b^	0.93 ± 0.04 ^b^	1.05 ± 0.11 ^b^	16.514	0.001
LZM 5 (μg/mL)	212.12 ± 6.31 ^c^	224.24 ± 6.43 ^c^	282.83 ± 2.67 ^a^	262.63 ± 2.67 ^b^	46.203	<0.001
LDH 6 (U/L)	1476.92 ± 106.59 ^a^	1219.38 ± 63.96 ^b^	1015.38 ± 53.29 ^b^	1083.08 ± 67.69 ^b^	7.275	0.011
TP 7 (g/L)	44.00 ± 0.66 ^c^	48.85 ± 0.82 ^b^	51.48 ± 0.35 ^a^	48.46 ± 0.60 ^b^	72.653	<0.001
ALB 8 (g/L)	18.83 ± 0.11 ^c^	19.46 ± 0.11 ^c^	22.74 ± 0.59 ^a^	21.47 ± 0.46 ^b^	22.41	<0.001
GLU 9 (mmol/L)	5.46 ± 0.02 ^a^	5.11 ± 0.11 ^a^	4.55 ± 0.17 ^b^	5.13 ± 0.03 ^a^	13.193	0.002
TG 10 (mmol/L)	6.07 ± 0.04 ^a^	5.9 ± 0.02 ^b,c^	5.69 ± 0.11 ^c^	5.94 ± 0.04 ^b^	19.006	0.001
T-CHO 11 (mmol/L)	10.14 ± 0.22	10.21 ± 0.17	9.75 ± 0.24	10.25 ± 0.37	2.552	0.129

Note: All the data are presented as mean ± SD (*n* = 3). Within the same row, different superscript letters indicate significant differences between the values (*p* < 0.05). ^1^ AKP: Alkaline phosphatase; ^2^ ACP: Acid phosphatase; ^3^ AST: Glutamic-oxalacetic transaminase; ^4^ ALT: Glutamic-pyruvic transaminase; ^5^ LZM: Lysozyme; ^6^ LDH: Lactate dehydrogenase; ^7^ TP: Total protein; ^8^ ALB: Albumin; ^9^ GLU: Glucose; ^10^ TG: Triglyceride; ^11^ T-CHO: Total cholesterol.

**Table 7:** The effects of *Rhodotorula mucilaginosa* in crayfish diets on the activity of antioxidant enzymes in the hemolymph.

Index	CK	RL	RM	RH	F-Value	*p*-Value
SOD ^1^ (U/mL)	106.98 ± 4.88 ^b^	127.21 ± 2.09 ^a^	126.05 ± 2.97 ^a^	119.3 ± 1.63 ^a^	8.685	0.007
CAT ^2^ (U/mL)	2.95 ± 0.12 ^c^	3.46 ± 0.08 ^b^	4.02 ± 0.03 ^a^	3.57 ± 0.15 ^b^	30.044	<0.001
GST ^3^ (U/mL)	15.09 ± 0.98 ^c^	20.35 ± 0.93 ^b^	27.37 ± 0.91 ^a^	22.98 ± 0.93 ^b^	30.044	<0.001
GPX ^4^ (U/mL)	360.63 ± 11.01 ^b^	401.68 ± 6.83 ^a^	416.84 ± 4.77 ^a^	365.68 ± 14.8 ^b^	7.334	0.011
MDA ^5^ (nmol/mL)	1.83 ± 0.07 ^a^	1.32 ± 0.07 ^b,c^	1.22 ± 0.12 ^c^	1.6 ± 0.09 ^a,b^	9.261	0.006
T-AOC ^6^ (mmol/L)	0.64 ± 0.03 ^c^	0.67 ± 0.01 ^b^	0.71 ± 0.01 ^a^	0.67 ± 0.04 ^b^	108.453	<0.001

Note: All the data are presented as mean ± SD (*n* = 3). Within the same row, different superscript letters indicate significant differences between the values (*p* < 0.05). ^1^ SOD: Superoxide dismutase; ^2^ CAT: Catalase; ^3^ GST: Glutathione S-transferase; ^4^ GPX: Glutathione peroxidase; ^5^ MDA: Malondialdehyde; ^6^ T-AOC: Total antioxidant capacity.

## Data Availability

The original contributions presented in the study are included in the article, and further inquiries can be directed to the corresponding author(s).
